# Supplementary material for: ANKRD1 is a mesenchymal-specific driver of cancer-associated fibroblast activation bridging androgen receptor loss to AP-1 activation
Source: Nat Commun. 2024 Feb 3;15:1038. doi: 10.1038/s41467-024-45308-w (PMC10838290; doi:10.1038/s41467-024-45308-w)
Supplement: Supplementary file 1 — Supplementary Information [file 41467_2024_45308_MOESM1_ESM.pdf]

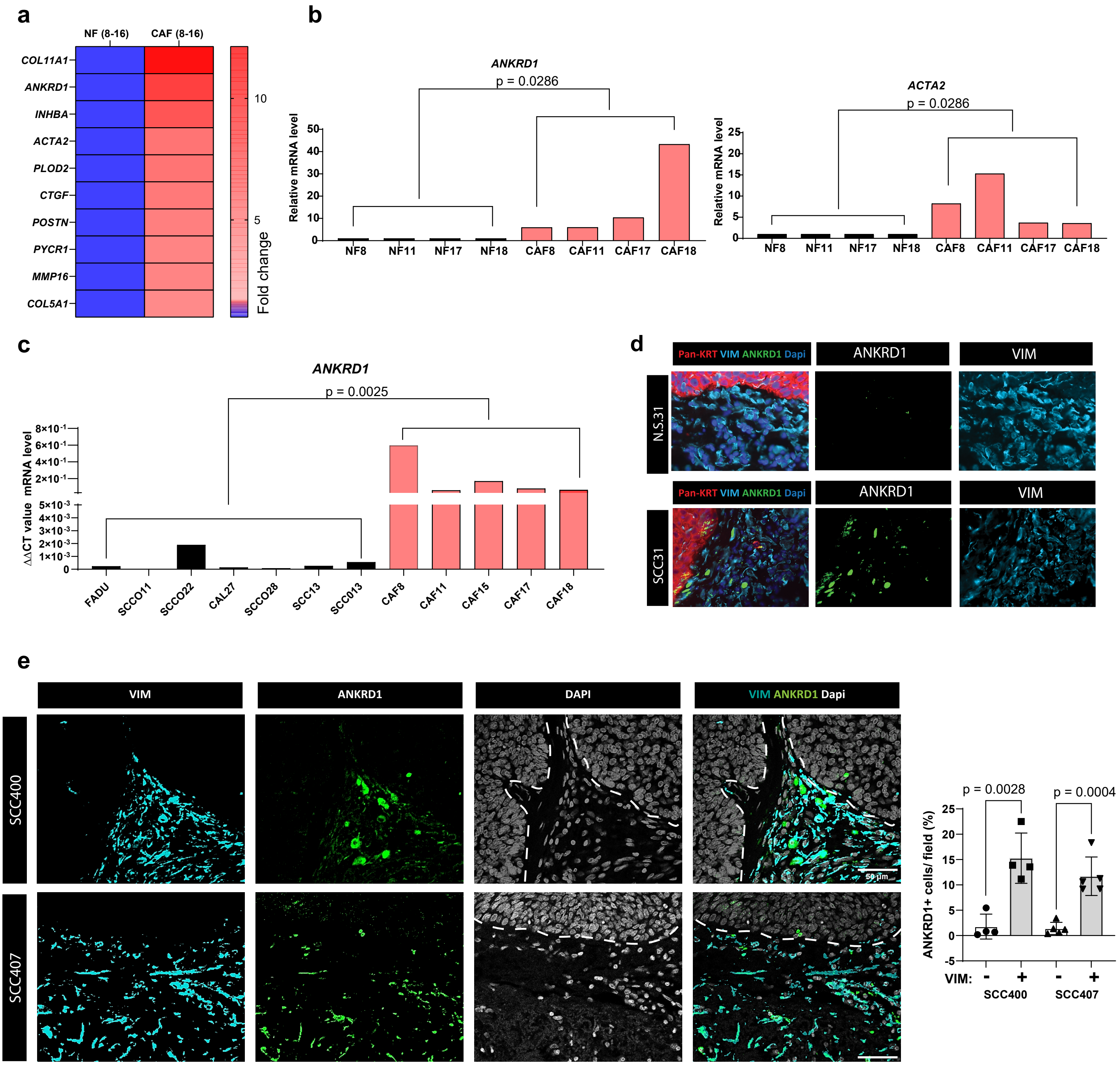

Supplementary Figure 1. ANKRD1 is a mesenchymal-specific cancer-associated fibroblast (CAF) marker.

(a) Heat map of changes in expression levels of the indicated CAF marker genes in CAF versus NF strains #8-16 as assessed by transcriptomic analysis (GSE122372) of the same RNA samples utilized for assessing levels of ANKRD1 in Fig. 1B. Blue versus red colour intensity depicts FC in the two groups, calculated by TAC software.

(b) RT-PCR analysis of ANKRD1 and ACTA2 expression in the indicated CAF versus matched NF strains at 1-2 later passages than those analysed in Fig. 1B. n (matched strains)=4, Two-tailed Mann Whitney test, *p=0.0286.

(c) mRNA expression analysis of ANKRD1 in multiple strains of squamous cell carcinomas (SCCs) and strains of CAFs. mRNA levels are expressed as amplification cycle thresholds normalized to RPLP0 (ΔΔCT), Kolmogorov-Smirnov test, n (strains)=7 for SCCs, and 5 for CAFs), **p=0.0025.

(d) Representative immunofluorescence analysis related to Fig. 1E using anti-ANKRD1 (green), anti-Vimentin (blue), and anti-Pan-KRT (red) antibodies in a skin SCC patient-derived sample and matched normal skin (NS). Scale bar: 100µM.

(e) IF analysis of two additional skin SCC samples besides that shown in Fig 1E with-ANKRD1 (green) and -vimentin (cyan) antibodies. Average ANKRD1 fluorescence signal intensity in vimentin-positive and negative cells in stromal versus tumor area, respectively, was quantified by ImageJ. n=2 (samples). n (fields per condition) > 4, two-tailed unpaired t-test, ** p=0.0028, *** p=0.0004.

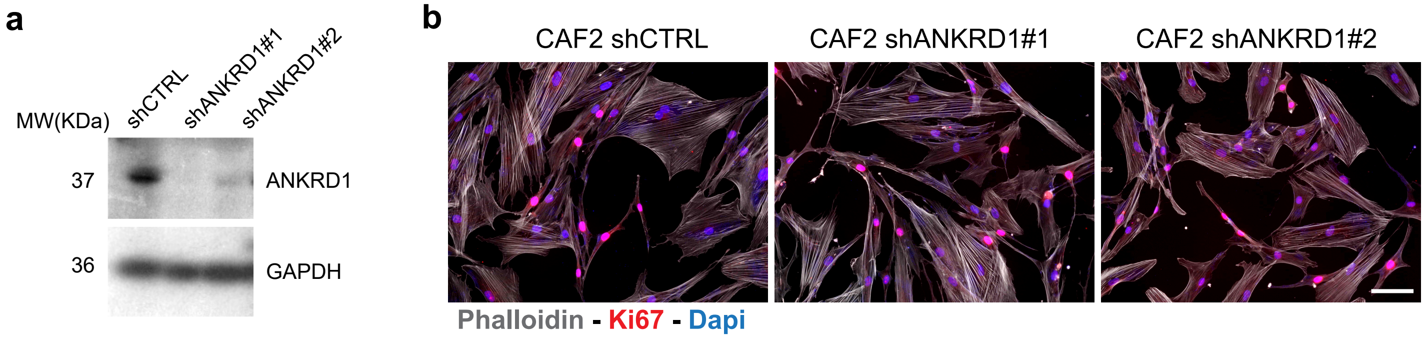

Supplementary Figure 2. ANKRD1 is required for cancer associated fibroblast (CAF) maintenance.

(a) Immunoblot analysis using anti ANKRD1 and GAPDH antibodies in CAF#1 infected with two shRNA targeting ANKRD1 (shANKRD1#1, shANKRD1#2) and shCTRL.

(b) IF images showing Ki67(red), phalloidin (gray) and DAPI (blue) in CAF#1 infected with two shRNA targeting ANKRD1 (shANKRD1#1, shANKRD1#2) and shCTRL. Quantification is shown in Fig 3B. Scale bar: 100μM.

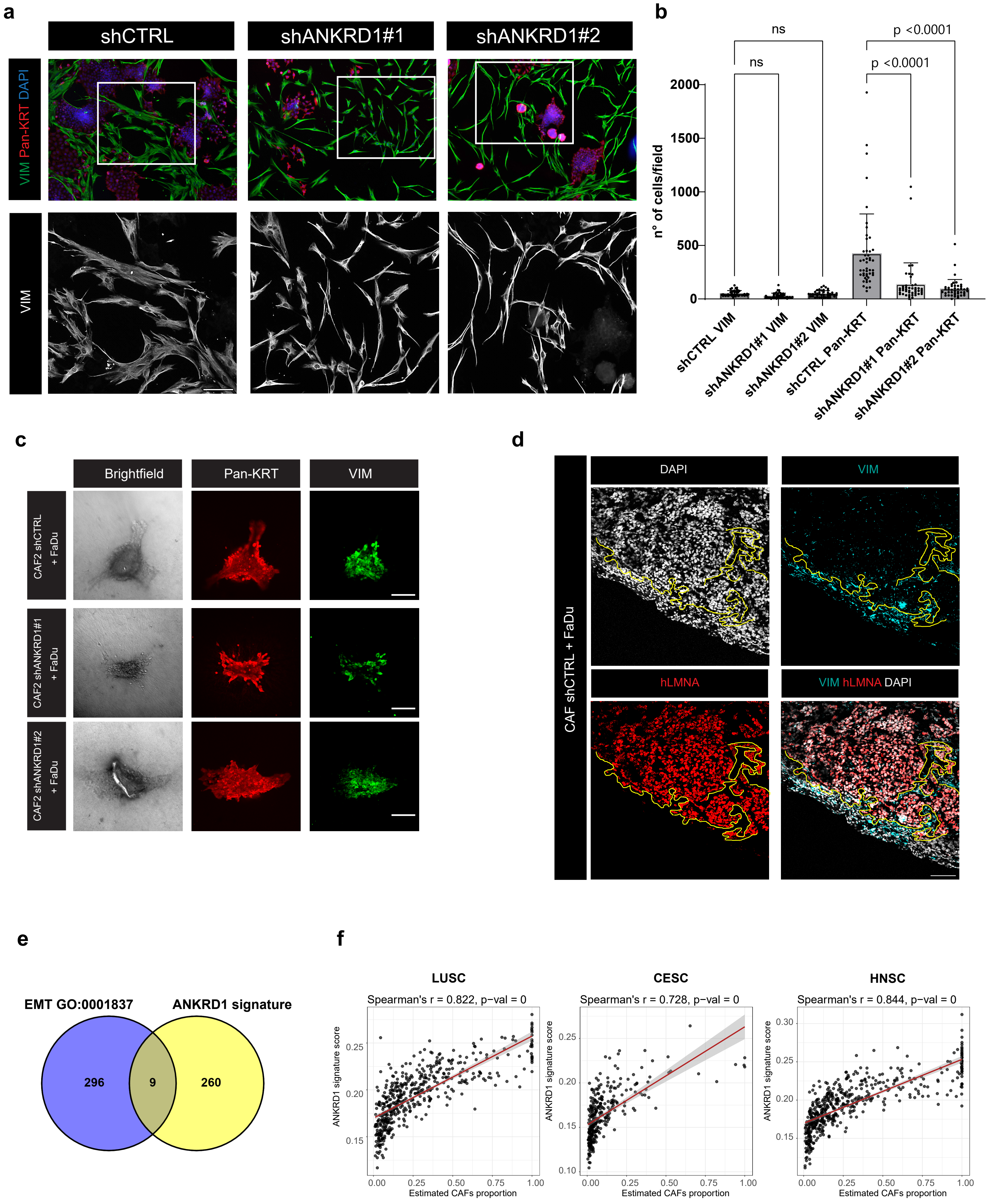
a
shCTRL
shANKRD1#1
shANKRD1#2
VIM Pan-KRT DAPI
VIM
b
ns
ns
p <0.0001
p <0.0001
n° of cells/field
2000
1500
1000
500
0
shCTRL VIM
shANKRD1#1 VIM
shANKRD1#2 VIM
shCTRL Pan-KRT
shANKRD1#1 Pan-KRT
shANKRD1#2 Pan-KRT
c
Brightfield
Pan-KRT
VIM
CAF2 shCTRL + FaDu
CAF2 shANKRD1#1 + FaDu
CAF2 shANKRD1#2 + FaDu
d
CAF shCTRL + FaDu
DAPI
VIM
hLMNA
VIM hLMNA DAPI
e
EMT GO:0001837
ANKRD1 signature
296
9
260
f
LUSC
Spearman's r = 0.822, p-val = 0
CESC
Spearman's r = 0.728, p-val = 0
HNSC
Spearman's r = 0.844, p-val = 0
ANKRD1 signature score
Estimated CAFs proportion
0.00
0.25
0.50
0.75
1.00
0.10
0.15
0.20
0.25
0.30

Figure S3. ANKRD1 regulates a CAF transcriptional program of clinical significance.

(a) Morphology of CAFs plus/minus ANKRD1 silencing by two shRNA vectors in cocultures with SCC cells. Double IF analysis with anti-vimentin and anti-keratin antibodies was used for cell type identification. Shown are representative images of vimentin-positive cells at low and high (single channel gray) magnification. Scale bar: 100μm

(b) Quantification of number of vimentin (VIM) and keratin (Pan-KRT) positive cells in co-cultures of SCC cells (Fadu) with ANKRD1-silenced CAFs by two different shRNA lentiviruses versus CAFs infected with a control vector (shCTRL), relative to Fig. 3c, exp 1-3 combined. Cell number determination was assessed by ImageJ software. n(fields/condition) >30; one-way Anova. VIM pLKO vs shANKRD#1: p=ns, VIM pLKO vs shANKRD#2: p=ns, PanK pLKO vs shANKRD#1: **** p <0.0001, PanK pLKO vs shANKRD#2: **** p<0.0001

(c) Additional representative images of double IF analysis of spheroids with anti-keratin and and-vimentin antibodies besides those shown in Fig. 3D. Scale bar: 200μm

(d) Venn diagram showing the overlap of ANKRD1 mesenchymal signature with a gene signature of epithelial-to-mesenchymal transition (EMT) (GO:0001837) downloaded from a collection of gene set, HARMONIZOME (https://maayanlab.cloud/Harmonizome/).

(e) Spearman's correlation analysis of ANKRD1 mesenchymal signature and CAF estimation. EPIC software (http://epic.gfellerlab.org/) was used to assess CAF proportion in the indicated tumor type (HNSCC, LUSC, CESC) from the TCGA consortium (https://portal.gdc.cancer.gov/). HNSCC: Spearman's r=0.844 (n=504); LUSC: Spearman's r=0.822 (n=485); CESC: Spearman's r=0.728 (n=297).

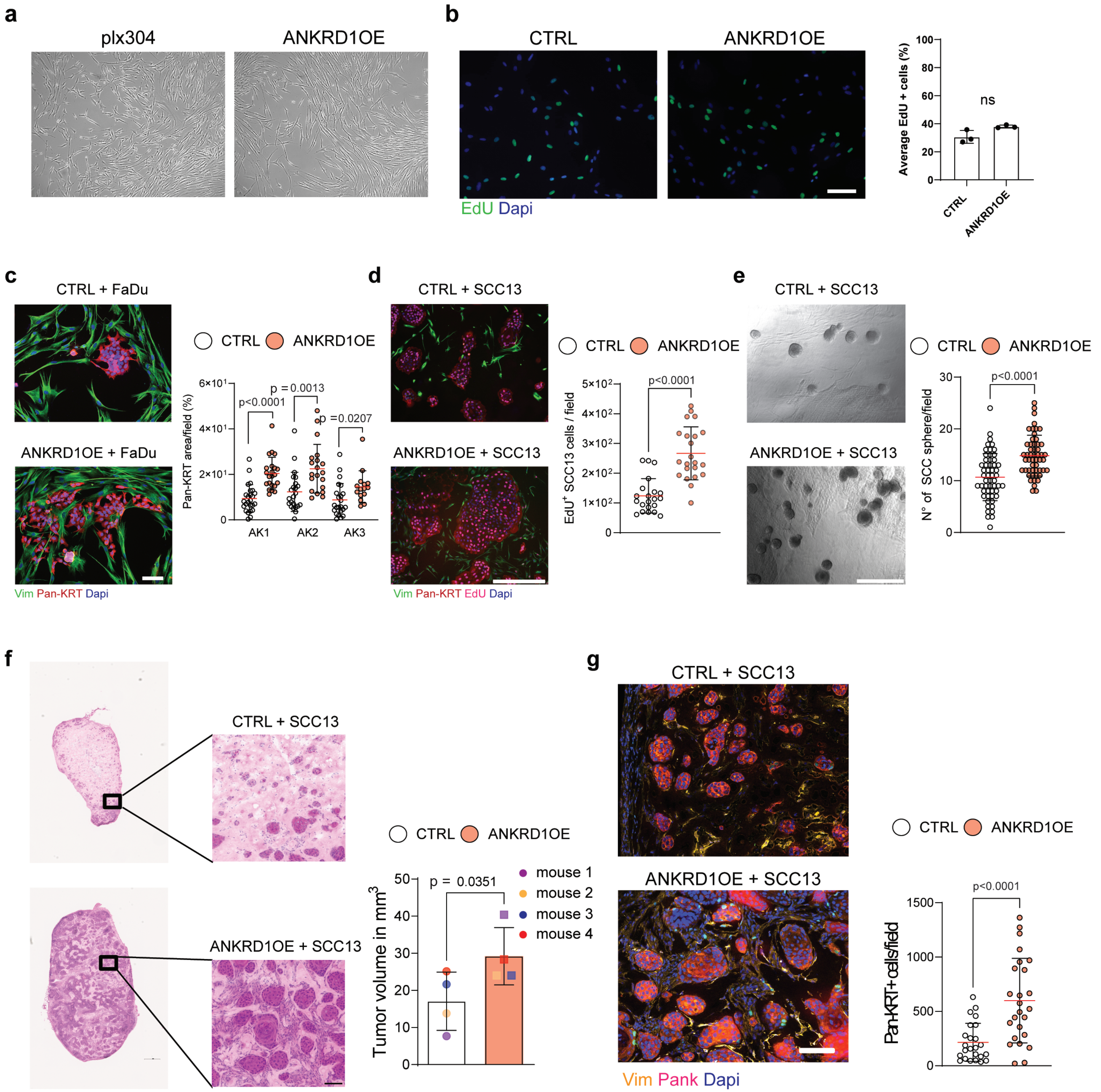
a
plx304
ANKRD1OE
b
CTRL
ANKRD1OE
EdU Dapi
Average EdU + cells (%)
ns
CTRL
ANKRD1OE
c
CTRL + FaDu
ANKRD1OE + FaDu
Vim Pan-KRT Dapi
CTRL ANKRD1OE
Pan-KRT area/field (%)
p<0.0001
p =0.0013
p =0.0207
AK1
AK2
AK3
d
CTRL + SCC13
ANKRD1OE + SCC13
Vim Pan-KRT EdU Dapi
CTRL ANKRD1OE
EdU+ SCC13 cells / field
p<0.0001
e
CTRL + SCC13
ANKRD1OE + SCC13
CTRL ANKRD1OE
N° of SCC sphere/field
p<0.0001
f
CTRL + SCC13
ANKRD1OE + SCC13
CTRL ANKRD1OE
Tumor volume in mm3
p = 0.0351
mouse 1
mouse 2
mouse 3
mouse 4
g
CTRL + SCC13
ANKRD1OE + SCC13
Vim Pank Dapi
CTRL ANKRD1OE
Pan-KRT+cells/field
p<0.0001

Figure S4. ANKRD1 is sufficient for converting HDFs to CAFs.

(a) Bright-field images of HDFs 5 days post infection and antibiotic selection (blasticidin) of HDFs with ANKRD1OE or CTRL vectors. Image processing was performed using Fiji/ImageJ (http://fiji.sc/Fiji). (b) Proliferation of fibroblasts infected with an ANKRD1 overexpressing vector (ANKRD1OE) or control vector (CTRL) was assessed by EdU incorporation assay. DAPI was used to stain the nuclei (blue), EdU signal is in green. Scale bar 100μM. Right panel: Quantification of the EdU signal. Mean±SD for three independent experiments (n° of replicates =3),(n° of fields/condition>10), two-tailed unpaired t-test, p=0.0538.

(c) Immunofluorescence analysis using anti-Pan-KRT (red) and anti-VIM (green) were used to identify FaDu cells and three HDF strains respectively. FaDu cells were co-cultured with either HDFs infected with ANKRD1OE or CTRL vectors for 5 days. Cells were admixed 1:1 ratio (1000 HDFs and 1000 FaDu). n=3 (strains). n (fields/strain/condition) >10, mean±SD, unpaired two-tailed t-test. AK1 **** p<0.0001, AK2 ** p=0.0013, AK3 * p=0.0207. Scale bar: 100μM.

(d) EdU labelling of co-cultured SCC13 cells. HDFs ANKRD1OE or CTRL were co-cultured for 5 days with SCC13 cells. Proliferation of SCC13 cells was assessed by EdU incorporation. Immunofluorescence analysis using anti-Pan-KRT (red), and anti-VIM (green) antibodies was used to identify SCC13 cells and HDFs respectively, EdU label (magenta). N° of replicates=3, mean±SD, unpaired two-tailed t-test **** p<0.0001. Scale bar: 500μm.

(e) Spheroid formation assay. HDFs were previously infected with ANKRD1OE or CTRL vectors. Next, HDFs were mixed with SCC13 cells and embedded in 100uL of Matrigel. After 5 days, the number of spheroids was quantified using ImageJ software. Scale bar: 500μM. Mean±SD, n=4 replicates, n (spheroids/condition) > 10, unpaired two-tailed T-test, **** p<0.0001.

(f) Representative images of H&E staining of back lesions formed by SCC13 cells co-injected with HDFs infected with either ANKRD1OE or CTRL vectors intradermally in contralateral mouse back skin. The cells were previously embedded in Matrigel before injection. Each panel has a representative zoom-in to visualize the cellular density of the cyst. Scale bar: 500μM, and 100μM (higher magnification). n (mice) =4, one-tailed unpaired t-test with Welch's correction, *p=0.0351.

(g) The increased SCC13 cell density and proliferation were quantified as the pan-keratin+ cells per field. N=4 mice. N≥2 low-magnification fields per lesion. Unpaired two-tailed t-test, **** p<0.001. Scale bar: 100μM.

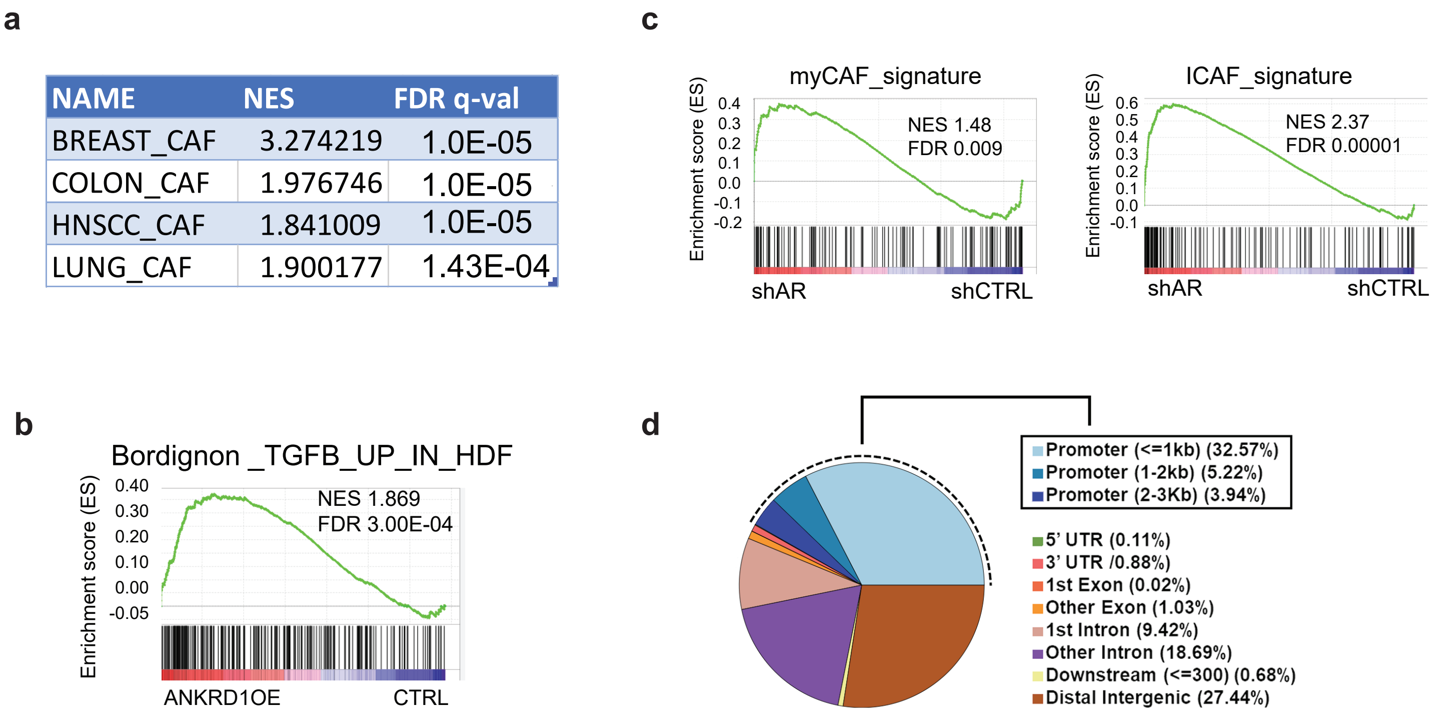

Figure S5. ANKRD1 is associated with myofibroblast-like CAFs.

(a) Gene Set Enrichment Analysis (GSEA) was performed using the Affymetrix expression profile of HDFs infected with ANKRD1OE or CTRL vectors. Gene sets derived from CAFs of HNSCC (GSE38517), colon (GSE44076), breast (https://doi.org/10.4161/cbt.7.8.6220), lung (GSE22874).

(b) GSEA analysis of TGFβ signature derived from GSE79621.

(c) Gene Set Enrichment Analysis (GSEA) was performed using myCAF and iCAF gene signatures (GSE93313) in the RNA-seq expression profile (GSE107321) of HDFs infected with two shRNAs targeting AR (shAR#1, #2) or one shRNA control vector (shCTRL). Enplots were used for graphical visualization. Normalized enrichment score (NES) and FDR were used for statistics.

(d) HDFs overexpressing ANKRD1 were processed for ChIP-seq analysis with an additional step of protein-protein crosslinking to pull down transcriptional complexes. ChIP was done using an anti-V5 antibody recognizing the V5-tag epitope expressed by ANKRD1 overexpressing HDFs. ChIPseeker analysis using Galaxy web tool (https://usegalaxy.org/) shows a visualization of ANKRD1 binding peak genomic coverage.

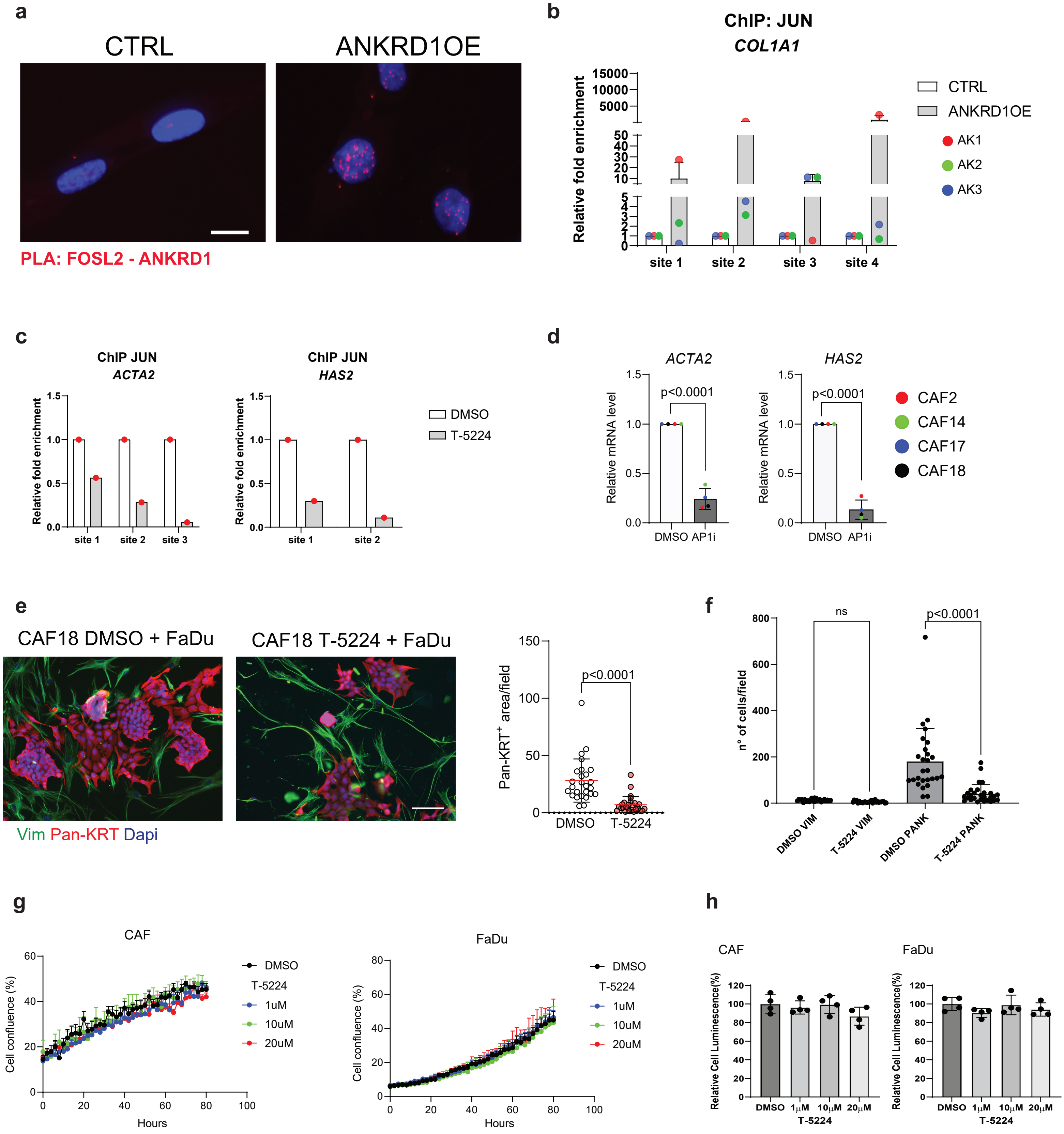
a
CTRL
ANKRD1OE
PLA: FOSL2 - ANKRD1
b
ChIP: JUN
COL1A1
Relative fold enrichment
15000
10000
5000
50
40
30
20
10
5
4
3
2
1
0
site 1
site 2
site 3
site 4
CTRL
ANKRD1OE
AK1
AK2
AK3
c
ChIP JUN
ACTA2
ChIP JUN
HAS2
Relative fold enrichment
1.5
1.0
0.5
0.0
site 1
site 2
site 3
DMSO
T-5224
d
ACTA2
HAS2
Relative mRNA level
p<0.0001
DMSO
AP1i
CAF2
CAF14
CAF17
CAF18
e
CAF18 DMSO + FaDu
CAF18 T-5224 + FaDu
Vim Pan-KRT Dapi
Pan-KRT+ area/field
150
100
50
0
p<0.0001
DMSO
T-5224
f
n° of cells/field
800
600
400
200
0
ns
p<0.0001
DMSO VIM
T-5224 VIM
DMSO PANK
T-5224 PANK
g
CAF
FaDu
Cell confluence (%)
60
40
20
0
80
Hours
0
20
40
60
80
100
DMSO
T-5224
1uM
10uM
20uM
h
CAF
FaDu
Relative Cell Luminescence(%)
120
100
80
60
40
20
0
DMSO
1μM
10μM
20μM
T-5224

Figure S6. ANKRD1 regulates CAF activation through AP-1 interaction

(a) Proximity ligation assay (PLA) with anti-ANKRD1 or anti-FOSL2 antibodies in HDFs cells infected with ANKRD1OE or CTRL vectors. Non-immune antibody (IgG) was used as negative control. Red fluorescence puncta from the juxtaposition of anti-ANKRD1 and anti-FOSL2 antibodies were visualized by confocal microscopy, with DAPI nuclear staining (blue). Shown are representative images and the number of puncta per cell, n° of cells>100 per condition. Scale bar is 20μM.

(b) ChIPmentation with anti-JUN antibody in HDFs infected with ANKRD1OE or CTRL vectors with qPCR amplification of indicated regions of the COL1A1 gene. Results are expressed as relative fold enrichment, relative to HDFs CTRL. Results were normalized using non-immune IgG, n =3 (strains).

(c) ChIPmentation with an anti-JUN antibody in HDFs treated with 20 μM of T-5224 or DMSO control for 48h. qPCR amplification of indicated regions for the ACTA2 and HAS2 genes. Results are expressed as relative enrichment fold relative to DMSO treated HDFs . Results were normalized using non-immune IgG. The experiment was performed once.

(d) RT-qPCR analysis of indicated genes in CAFs treated with 20 μM of T-5224 or DMSO for 48h. Values are expressed relative to DMSO; mRNA levels are expressed as amplification cycle thresholds normalized to RPLP0. N = 4 (strains), mean±SD, unpaired two-tailed t-test, ****p<0.0001.

(e) Double-immunofluorescence analysis using anti-Pan-KRT (red) and anti-VIM (green) were used to identify FaDu cells and CAFs respectively. FaDu cells were co-cultured with either CAFs pre-treated for 72 hours with 20 μM of T-5224 or DMSO, and the co-culture was carried out for 7 days. Cells were admixed 1:1 ratio (1000 CAFs and 1000 FaDu). Quantification was assessed by measuring Pan-KRT positive area/ field by ImageJ. n (fields/condition) >1, mean±SD, unpaired two-tailed t-test, **** p<0.0001. Scale bar: 100μM.

(f) Quantification of number of vimentin (VIM) and keratin (Pan-KRT) positive cells in co-cultures of SCC cells (Fadu) with CAFs pre-treated with 20 μM of T-5224 versus DMSO control relative to Supplementary Fig 6E. Quantification was determined by ImageJ cell counting feature. n (fields/condition) >20; one-way Anova with Holm-Šídák's multiple comparisons test, VIM: DMSO vs T-5224 p=ns, PanK: DMSO vs T-5224 ****, p<0.0001.

(g) Proliferation by live-cell imaging assays (IncuCyte) of CAFs treated with 1, 10, and 20 μM of T-5224 versus DMSO control. Cells were plated in 96-well plates followed by cell density measurements (four images per well every 2 h for 168 h).Error bars indicate 4 technical replicates. Mean±SD.The experiment was performed once.

(h) CellTiter-Glo luminescence assay of CAF proliferation +/- treatment with 1, 10, and 20 μM of T-5224 versus DMSO control. Cells were plated in 96-well plates followed by luminescence intensity measurements. Cell were plated at low density (1000 cell/well) and the experiment was stopped after 7 days. Dots indicate 4 technical replicates. Mean±SD. The experiment was performed once.

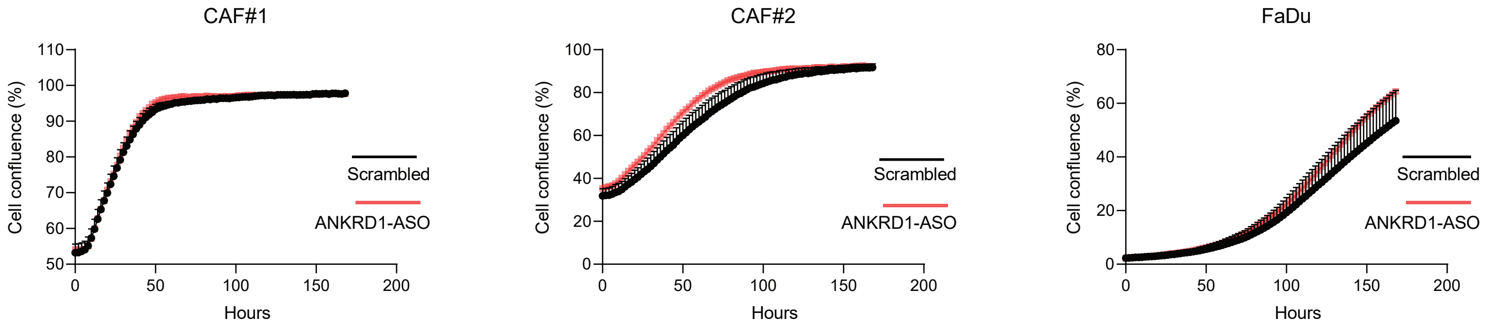

Supplementary Fig. 7. ANKRD1 targeting by ASO has no effect on either CAFs or SCC cell proliferation

Proliferation by live-cell imaging assays (IncuCyte) of CAFs (CAF#1, CAF#2) or SCC cells (FaDu) transfected with 100nM of ANKRD1-ASO or Scrambled-ASO. Cells were plated in 96-well plates followed by cell density measurements (four images per well every 2 h for 168 h). Error bars indicate 4 technical replicates. Mean±SD. The experiment was performed once.

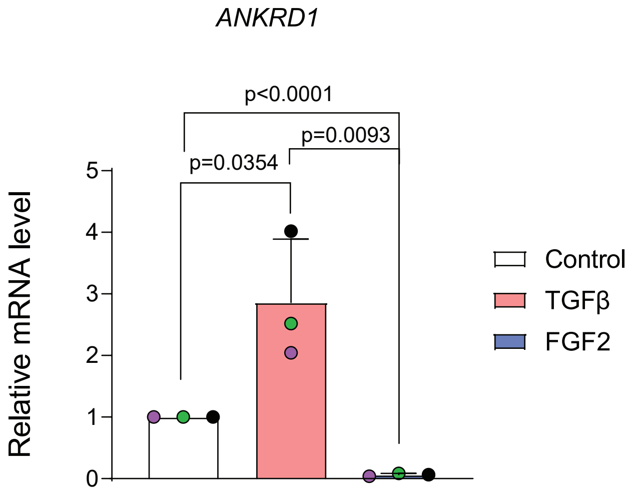

Figure S8. Opposite modulation of ANKRD1 by TGFβ and FGF2 stimulation.

RT-qPCR analysis of ANKRD1 gene in HDFs treated with 10 ng of TGFβ, FGF2, or vehicle control (Et-OH) for 48h. Values are expressed relative to Control; mRNA levels are expressed as amplification cycle thresholds normalized to 36ß4. n (strains)=3, mean±SD, unpaired two-tailed t-test, * p=0.0354, **p=0.0093, ****p<0.0001.
