# Supplementary material for: ANKRD1 is a mesenchymal-specific driver of cancer-associated fibroblast activation bridging androgen receptor loss to AP-1 activation
Source: Nat Commun. 2024 Feb 3;15:1038. doi: 10.1038/s41467-024-45308-w (PMC10838290; doi:10.1038/s41467-024-45308-w)
Supplement: Supplementary file 3 — Description of Additional Supplementary Files [file 41467_2024_45308_MOESM3_ESM.pdf]

Description of Additional Supplementary Files

File name: Supplementary Data 1

Description: List of transcription factors upregulated in CAFs versus matched HDFs, in shAR versus shCTRL, and downmodulated in CAFs treated with JQ1

File name: Supplementary Data 2

Description: List of genes downmodulate or upregulated in 4 different ANKRD1-silenced CAFs with 2 different shRNA against ANKRD1

File name: Supplementary Data 3

Description: ANKRD1 mesenchymal gene signature derived from the transcriptomic profile of ANKRD1-silenced CAFs crossed with cell type specific gene expression

File name: Supplementary Data 4

Description: List of genes downmodulate or upregulated in 3 different ANKRD1-overexpressed HDFs

File name: Supplementary Data 5

Description: Genes belonging to TGFβ signaling or IFN signaling enriched in ANKRD1OE up regulated genes olverlapping with shAR upregulated genes, or in CTRL genes overlapping with shAR upregulated genes

File name: Supplementary Data 6

Description: Transcription factor family enriched in ANKRD1 ChIPseq

File name: Supplementary Data 7

Description: Expression levels of AP-1 family members in CAFs

File name: Supplementary Data 8

Description: List of reagents used for this manuscript
