# Supplementary material for: ANKRD1 is a mesenchymal-specific driver of cancer-associated fibroblast activation bridging androgen receptor loss to AP-1 activation
Source: Nat Commun. 2024 Feb 3;15:1038. doi: 10.1038/s41467-024-45308-w (PMC10838290; doi:10.1038/s41467-024-45308-w)
Supplement: Supplementary file 12 — Reporting Summary [file 41467_2024_45308_MOESM12_ESM.pdf]

nature portfolio

Corresponding author(s): Paolo Dotto

Last updated by author(s): Nov 16, 2023

Reporting Summary

Nature Portfolio wishes to improve the reproducibility of the work that we publish. This form provides structure for consistency and transparency in reporting. For further information on Nature Portfolio policies, see our Editorial Policies and the Editorial Policy Checklist.

Statistics

For all statistical analyses, confirm that the following items are present in the figure legend, table legend, main text, or Methods section.

n/a	Confirmed	
[ ]	[x]	The exact sample size (*n*) for each experimental group/condition, given as a discrete number and unit of measurement
[ ]	[x]	A statement on whether measurements were taken from distinct samples or whether the same sample was measured repeatedly
[ ]	[x]	The statistical test(s) used AND whether they are one- or two-sided<br>*Only common tests should be described solely by name; describe more complex techniques in the Methods section.*
[x]	[ ]	A description of all covariates tested
[ ]	[x]	A description of any assumptions or corrections, such as tests of normality and adjustment for multiple comparisons
[ ]	[x]	A full description of the statistical parameters including central tendency (e.g. means) or other basic estimates (e.g. regression coefficient) AND variation (e.g. standard deviation) or associated estimates of uncertainty (e.g. confidence intervals)
[ ]	[x]	For null hypothesis testing, the test statistic (e.g. *F*, *t*, *r*) with confidence intervals, effect sizes, degrees of freedom and *P* value noted<br>*Give P values as exact values whenever suitable.*
[x]	[ ]	For Bayesian analysis, information on the choice of priors and Markov chain Monte Carlo settings
[x]	[ ]	For hierarchical and complex designs, identification of the appropriate level for tests and full reporting of outcomes
[ ]	[x]	Estimates of effect sizes (e.g. Cohen's *d*, Pearson's *r*), indicating how they were calculated

Our web collection on statistics for biologists contains articles on many of the points above.

Software and code

Policy information about availability of computer code

Data collection	All the data used in the manuscript was obtained from publicly available depositories and websites, including GEO and MSigDB. The GEO numbers are specified in<br>Methods section of the manuscript. Light Cycler 480 (Roche) associated software, ZEISS LSM880 confocal microscope, and NanoZoomer 560 microscope<br>associated softwares Nikon Ndp.View2. Fiji/ImageJ.
Data analysis	clusterProfiler 4.0<br>Seurat 4.1.1<br>GSEA 4.1.0<br>R package Limma<br>R package singscore v1.0.0 R package EPIC v1.1.5<br>R package survival v2.43-3 Enrichr<br>Graph Pad Prism Software IGV Software<br>Microsoft Excel 365<br>PeaksAnnotator (HOMER)<br>Bowtie2 Version 2.3.0<br>MACS<br>the Burrows-Aligner (BWA) 70 PeaksAnnotator<br>(HOMER)<br>Bowtie2 Version 2.3.0

Microsoft Excel 365

For manuscripts utilizing custom algorithms or software that are central to the research but not yet described in published literature, software must be made available to editors and reviewers. We strongly encourage code deposition in a community repository (e.g. GitHub). See the Nature Portfolio guidelines for submitting code & software for further information.

Data

Policy information about availability of data

All manuscripts must include a data availability statement. This statement should provide the following information, where applicable:

- Accession codes, unique identifiers, or web links for publicly available datasets
- A description of any restrictions on data availability
- For clinical datasets or third party data, please ensure that the statement adheres to our policy

GSE22862 (https://www.ncbi.nlm.nih.gov/gds/?term=GSE22862)
GSE29270 (https://www.ncbi.nlm.nih.gov/geo/query/acc.cgi?acc=GSE29270)
GSE46824 (https://www.ncbi.nlm.nih.gov/geo/query/acc.cgi?acc=GSE46824)
GSE38517 (https://www.ncbi.nlm.nih.gov/geo/query/acc.cgi?acc=GSE38517)
E-MTAB-2509 (https://www.ebi.ac.uk/biostudies/arrayexpress/studies/E-MTAB-2509)
GSE40839 (https://www.ncbi.nlm.nih.gov/geo/query/acc.cgi?acc=GSE40839)
GSE103322 (https://www.ncbi.nlm.nih.gov/geo/query/acc.cgi?acc=GSE103322)
TCGA (https://portal.gdc.cancer.gov/)
GSE218198 (https://www.ncbi.nlm.nih.gov/geo/query/acc.cgi?acc=GSE218198)
GSE218214 (https://www.ncbi.nlm.nih.gov/geo/query/acc.cgi?acc=GSE218214)
GSE218204 (https://www.ncbi.nlm.nih.gov/geo/query/acc.cgi?acc=GSE218204)
GSE107320 (https://www.ncbi.nlm.nih.gov/geo/query/acc.cgi?acc=GSE107320)
GSE135893 (https://www.ncbi.nlm.nih.gov/geo/query/acc.cgi?acc=GSE135893)
GSE81406 (https://www.ncbi.nlm.nih.gov/geo/query/acc.cgi?acc=GSE81406)

Research involving human participants, their data, or biological material

Policy information about studies with human participants or human data. See also policy information about sex, gender (identity/presentation), and sexual orientation and race, ethnicity and racism.

Reporting on sex and gender	Discarded unidentified adult human tissue from skin squamous cell carcinomas for research and from healthy human foreskin
Reporting on race, ethnicity, or other socially relevant groupings	N/A
Population characteristics	N/A
Recruitment	N/A
Ethics oversight	For CAFs: Institutional Review Board (IRB# 2018P003156), Massachusetts General Hospital, Boston<br>For fibroblasts: Samples were obtained from the surgery department of the Centre hospitalier universitaire vaudois (CHUV, Switzerland), with patient and institution consent (UNIL; protocol # 222-12)

Note that full information on the approval of the study protocol must also be provided in the manuscript.

Field-specific reporting

Please select the one below that is the best fit for your research. If you are not sure, read the appropriate sections before making your selection.

☒ Life sciences ☐ Behavioural & social sciences ☐ Ecological, evolutionary & environmental sciences

For a reference copy of the document with all sections, see nature.com/documents/nr-reporting-summary-flat.pdf

Life sciences study design

All studies must disclose on these points even when the disclosure is negative.

Sample size	Sample size was determined using power calculation allowing the use of statistical analysis.<br>Statistical significance of differences between experimental groups and controls was assessed by two-tailed unpaired or paired t-test, and one way AN OVA, as indicated in the legends. In all cases, p values< 0.05 were considered as statistically significant.<br>In animal experiments, we used a power calculation to determine the minimum sample size that is needed in order to have sufficient statistical power to detect a treatment effect (20-30% change in tumor growth).
Data exclusions	No data or sample were excluded from the analysis.
Replication	To verify reproducibility of experimental findings multiple biological strains and technical replicates were used throughout the study. All the

	replicates (number and strains are reported in the figure legends for each experiment).All attempts were successful. No cell line or data were excluded.
Randomization	In all the experiments, the animals were injected contralaterally with both control and treated cells. In the gavage experiments the animals were randomly assigned to control or treated group.
Blinding	The researchers involved in the study were not blinded during sample obtainment or data analysis. Considering the multiple aspects of the study, complete blinding of the investigators was not possible for the data collection, nor relevant considering the cross-check of the results.<br>In vitro experiments and bioinformatic data analysis were cross checked by the Investigators involved in the study.<br>The in vivo experiments were carried out as a team.

Reporting for specific materials, systems and methods

We require information from authors about some types of materials, experimental systems and methods used in many studies. Here, indicate whether each material, system or method listed is relevant to your study. If you are not sure if a list item applies to your research, read the appropriate section before selecting a response.

Materials & experimental systems

n/a	Involved in the study
☐	☒ Antibodies
☐	☒ Eukaryotic cell lines
☒	☐ Palaeontology and archaeology
☐	☒ Animals and other organisms
☒	☐ Clinical data
☒	☐ Dual use research of concern
☒	☐ Plants

Methods

n/a	Involved in the study
☐	☒ ChIP-seq
☒	☐ Flow cytometry
☒	☐ MRI-based neuroimaging

Antibodies

Antibodies used	A full list of primary and secondary antibodies, including catalog numbers, clone names and dilutions, used in the study<br>is provided in the Supplementary Data file 8.<br>Antibodies SOURCE Catalog number RRID Assays and dilution used<br>Rabbit monoclonal anti-AR (D6F11) Cell Signaling Technology Cat# 5153S AB_10691711 WB (1:1000)<br>Rabbit polyclonal anti-GAPDH (FL-335) Santa Cruz Biotechnology Cat# sc-25778 AB_10167668 WB (1:10000)<br>Rabbit polyclonal V5 tag antibody GeneTex Cat# GTX117997 AB_10618481 ChIP (1:200)<br>Rabbit polyclonal Ki67 antibody GeneTex Cat# GTX20833 AB_367700 IF (1:300)<br>Mouse monoclonal Antibody to Human Cytokeratin (Pan) BMA Biomedicals Cat# T-1302 AB_1227343 IF(1:300)<br>Rat monoclonal Antibody to Human Vimentin R&D System Cat# MAB2105 AB_2241653 IF (1:300)<br>Mouse monoclonal to Human ANKRD1 (G-2) Santa Cruz Biotechnology Cat# sc-365056 AB_10844192 PLA (1:100), WB (1:200), ChIP (5µg)<br>Rabbit polyclonal anti-ANKRD1 Abcam Cat# ab247009 N/A IF (1:100)<br>Rabbit monoclonal anti-JUN (60A8) Cell Signaling Technology Cat# 9165 AB_2130165 WB (1:1000), ChIP (5µg), PLA (1:100), IF (1:200)<br>Rabbit monoclonal anti-FOSL2 Cell Signaling Technology Cat# 19967 AB_2722526 WB (1:1000), PLA (1:100)<br>Rabbit polyclonal anti-FOS Cell Signaling Technology Cat# 4384 AB_2106617 WB (1:1000)<br>Anti-V5-tag mAb-Magnetic Beads (Monoclonal Antibody) MBL Cat# M167-11 AB_10795284 IP (30µL), ChIP (30µL)<br>Anti-LMNA mAB Abcam CAT# ab108595 AB_10866185 IF (1:200)<br>Mouse monoclonal anti-JUN (G2) Santa Cruz Biotechnology Cat# sc-74543 AB_1121646 PLA (1:100)<br>Glutathione Magnetic Agarose Beads Pierce - ThermoFisher Cat# 78601 N/A Co-IP (15µL)<br>Goat anti-rabbit IgG HRP Promega Cat# W401B AB_430833 WB (1:10000)<br>Goat anti-mouse IgG HRP Promega Cat# W4021 AB_430834 WB (1:10000)<br>Donkey anti-mouse IgG Alexa Fluor™ 488 ThermoFisher Cat# A-21202 AB_141607 IF (1:1000)<br>Donkey anti-rabbit IgG Alexa Fluor™ 568 ThermoFisher Cat# A-10042 AB_2534017 IF (1:1000)<br>Donkey anti-goat IgG Alexa Fluor™ 647 ThermoFisher Cat# A-21447 "AB_2535864" IF (1:1000)<br>Chicken anti-Rat IgG Alexa Fluor™ 647 ThermoFisher Cat# A-21472 AB_2535875 IF (1:1000)<br>Donkey anti-rabbit IgG Alexa Fluor™ 488 ThermoFisher Cat# A-21206 "AB_2762833" IF (1:1000)<br>Donkey anti-mouse IgG Alexa Fluor™ 568 ThermoFisher Cat# A10037 AB_2534013 IF (1:1000)<br>Chicken anti-Rabbit IgG Alexa Fluor™ 647 ThermoFisher Cat# A-21443 AB_2535861 IF (1:1000)<br>Donkey anti-goat IgG Alexa Fluor™ 488 ThermoFisher Cat# A-11055 AB_2534102 IF (1:1000)
Validation	All antibodies underwent an application-specific validation by the companies. We indicate the Research Resource Identifier (https://scicrunch.org/resources), providing a link to the validation and previous published use of each antibody.

Eukaryotic cell lines

Policy information about cell lines and Sex and Gender in Research

Cell line source(s)	A description of the different squamous cell carcinoma cell lines and primary fibroblasts and CAFs is provided in the matherial and methods section and in figure legends<br>Cancer cell lines:<br>SCC13 RRID:CVCL_4029, Fadu RRID:CVCL_1218, SCCO22 RRID:CVCL_7731,SCCO13 RRID:CVCL_C06, Cal27 RRID:CVCL_1107 SCCO28 RRID:CVCL_7738, 293T ATCC RRID:CVCL_0063<br>Primary Fibroblasts and CAFs discarded skin: CAF#1, CAF#, CAF#3, CAF#7, CAF#8, NF#8, CAF#9, NF#9, CAF#10, NF#10, CAF#11, NF#11, CAF#12, NF#12, CAF#13 NF#13, CAF#14, NF#14, CAF#15, NF#15, CAF#16 NF#16, CAF#17, NF#17, CAF#18, NF#18, HFF, PB1, AK1, AK2, AK3, GB4, TP45, GP181
Authentication	Primary CAF and fibroblasts were derived from discarded healthy donor skin and were tested by IF to be vimentin positive and Keratin-negative.<br>All SCC cell lines were commercially available and we indicated the Research Resource identifier linking to previous publications. Morphology, presence of IF markers (keratins, positive), the presence of keratinocyte-specific gene by PCR with human-specific primers were used for validation. New ATTC purchased vials was used for 293T cells.
Mycoplasma contamination	All cell lines were routinely checked for the absence of mycoplasma by HOECHST staining without permeabilization of parallel cultures. Observation at high magnification {63x) showed that all were negative.
Commonly misidentified lines<br>(See ICLAC register)	No commonly misindentified cell strains were used

Animals and other research organisms

Policy information about studies involving animals; ARRIVE guidelines recommended for reporting animal research, and Sex and Gender in Research

Laboratory animals	Mouse: NOD.CB17-Prkdcscid/J (6-8 weeks) Jackson laboratory
Wild animals	No wild animals were used.
Reporting on sex	The experiments were performed in both female and male mice.
Field-collected samples	No field collected samples were used in the study.
Ethics oversight	All animal studies were carried out according to Swiss guidelines for the use of laboratory animals, with protocols approved by the University of Lausanne animal care and use committee and the veterinary office of Canton Vaud (animal license No. 1854.4f/1854.5a).

Note that full information on the approval of the study protocol must also be provided in the manuscript.

Plants

Seed stocks	*Report on the source of all seed stocks or other plant material used. If applicable, state the seed stock centre and catalogue number. If plant specimens were collected from the field, describe the collection location, date and sampling procedures.*
Novel plant genotypes	*Describe the methods by which all novel plant genotypes were produced. This includes those generated by transgenic approaches, gene editing, chemical/radiation-based mutagenesis and hybridization. For transgenic lines, describe the transformation method, the number of independent lines analyzed and the generation upon which experiments were performed. For gene-edited lines, describe the editor used, the endogenous sequence targeted for editing, the targeting guide RNA sequence (if applicable) and how the editor was applied.*
Authentication	*Describe any authentication procedures for each seed stock used or novel genotype generated. Describe any experiments used to assess the effect of a mutation and, where applicable, how potential secondary effects (e.g. second site T-DNA insertions, mosiacism, off-target gene editing) were examined.*

ChIP-seq

Data deposition

- [x] Confirm that both raw and final processed data have been deposited in a public database such as GEO.
- [x] Confirm that you have deposited or provided access to graph files (e.g. BED files) for the called peaks.

Data access links<br>*May remain private before publication.*	GEO accession https://www.ncbi.nlm.nih.gov/geo/query/acc.cgi?acc=GSE218204

Files in database submission	BEDGRAPH
Genome browser session (e.g. UCSC)	Bedgraphs are provided in the GEO to allow visualization in IGV.

Methodology

Replicates	GSM6736617 Anti-ANKRD1 antibody<br>GSM6736618 Input
Sequencing depth	GSM6736617 Anti-ANKRD1 antibody = Total Sequences 29614286 = Run type : HiSeq 4000 SR 150 cycles<br>GSM6736618 Input = Total Sequences 17856238 = Run type : HiSeq 4000 SR 150 cycles
Antibodies	Rabbit polyclonal V5 tag antibody, GeneTex Cat# GTX117997
Peak calling parameters	FASTA files were aligned using the Burrows-Wheeler<br>Aligner (BWA) 70, and MACS2 software was used to identify narrow peaks with a q-value cut-off of 0.05.Peaks were annotated and merged using the annotatePeaks.pl and mergepeaks.pl functions from the HOMER software.<br>q-value cut-off of 0.05 was used, and the following number of peaks was obtained:Experiment IP/Input Count of narrow peaks= 40745
Data quality	First step: check the raw data quality using FastQC.<br>The procedure for data trimming is in the following:<br>(1) Discard the reads with low quality (proportion of low quality bases larger than 50%);<br>(2) Discard the reads with N ratio (unsure base) larger than 15%;<br>(3) Discard the reads with adaptor at the 5'-end;<br>(4) Discard the reads without adaptor and inserted fragment at the 3'-end;<br>(5) Trim the adapter sequence at the 3'-end;<br>(6) Discard the reads whose length are less than 18nt after trimming.
Software	Raw data files from ChIP-seq assays were aligned to the GRCh38 genome with Bowtie2 Version 2.3.0 (http://bowtie-bio.sourceforge.net/bowtie2). Duplicates were removed with Picard (https://broadinstitute.github.io/picard/) and, for peak detection, MACS2 software (http://liulab.dfci.harvard.edu/MACS) was used with a p-value cutoff of 1.00e–04. Peaks were annotated with HOMER (http://homer.ucsd.edu/homer/index.html). The Integrative Genomics Viewer (http:// software.broadinstitute.org/software/igv/) was used for graphic illustration of ChIP-seq peaks.
